# Influence of length, diameter and position of the implant in its fracture incidence: A Systematic Review

**DOI:** 10.15171/joddd.2019.017

**Published:** 2019-08-14

**Authors:** Marcelo Coelho Goiato, Agda Marobo Andreotti, Daniela Micheline dos Santos, Adhara Smith Nobrega, Fernanda Pereira de Caxias, Lisiane Cristina Bannwart

**Affiliations:** ^1^Professor, Department of Dental Materials and Prosthodontics; Araçatuba Dental School, São Paulo State University Júlio de Mesquita Filho, UNESP, Araçatuba, Brazil; ^2^PhD Student; Department of Dental Materials and Prosthodontics; Araçatuba Dental School, São Paulo State University Júlio de Mesquita Filho, UNESP, Araçatuba, Brazi; ^3^Professor, Department of Dental Materials and Prosthodontics, Manaus, UNIP, Brazil

**Keywords:** Dental implants, dental restoration failure, mouth rehabilitation, periprosthetic fractures.

## Abstract

***Background.*** Implant fractures can cause difficult problems for patients and dentists. This systematic review aimed to determine the influence of some implant parameters on the occurrence of their fracture and to determine the incidence of fractures reported in recent years.

***Methods.*** A search was conducted in Pubmed database, from which 12 studies published in the last 12 years were selected.

***Results.*** This review reported a 2% incidence of implant fracture. Most implants had been in function between 3 and 4 years until fracture. The studies did not provide necessary information to establish a relationship between the different parameters of implants and the incidence of fractures.

***Conclusion.*** Thus, the indication of type, diameter and length of an implant and the bone quality in the region receiving it should be studied and accurately examined for each individual case in order to avoid future failures.

## Introduction

**S**ince the beginning of implantology, implant-supported prostheses have been a highly predictable treatment for total or partial edentulous patients.^1,2^ However, complications affecting implant osseointegration can occur in specific situations, and professionals should be aware of treatment limitations to avoid risky situations that could lead to implant and prosthesis failures.^2,3^ Implantologyis constantly seeking improvements in and development of new materials and implants. Titanium implants have been used for decades and the implants fabricated with zirconia were developed as an alternative to some problems caused by titanium.^4^

Complications related to dental implants can be classified as biological or mechanical. Although mechanical complications are rare, they can lead to serious clinical consequences.^5^ These complications might involve loosening or fracture of the prosthetic screw, loosening or fracture of the abutment screw, and also implant fracture.^2^ Recent studies have investigated the rates, pattern and potential risk of implant fractures.^6-8^ Lee et al found lower risk of fractures in implants with wide‐diameter microthreads, placed in patients with a history of bone graft, as well as those positioned in the mandibular anterior area.^6^ Karl, Scherg and Grobecker-Karl reported that torque might be a risk factor for fracture in zirconia implants.^8^

However, the literature reports a low incidence of implant fractures, but when it occurs, it can cause difficult problems for patients and dentists. Determining the etiology of fractures can be a challenge for dentists.^9^ A great number of factors should be considered when analyzing the possible causes for dental implant fractures. One of the main causes is the biomechanical overloading that can occur due to parafunctional activities such as bruxism, malocclusion, presence of cantilevers and lack of passive fit of implant-supported prostheses, resulting in fatigue.^10-12^ Also, the implant location, insufficient quantity of implants supporting the prosthesis, implant material, implant diameter and other factors must also be considered.^13^ The treatment of a fractured implant can be a challenge for the clinician due to surgical, rehabilitative and emotional implications.^12^

Many factors can influence implant fractures and the complexity of a failure on this level, regarding both the unsuccessful treatment and the failure resolution. This systematic review aimed to evaluate the effect of some implant parameters, such as length, diameter and position, on the occurrence of fracture and to determine the incidence of fractures reported in recent years.

## Methods

### Search Strategy and Screening of Articles

An electronic survey was conducted in Pubmed database using the filters: Species (Humans) and Languages (English). Two examiners using the following term “dental implant fracture” conducted the search process independently. The search period was the interval from 07/31/2004 to 03/02/2016.

Study selection was initially directed to title and abstract analysis. Given the existence of few randomized controlled studies, it included prospective and retrospective studies.

Subsequently, the eligible studies were analyzed and included or excluded from the total sample. Thus, the population, intervention, comparison and outcome (PICO), as recommended by PRISMA,^13^ were determined as questioning criteria to organize a clear clinical question and an appropriate inclusion approach where the "Population" corresponds to patients rehabilitated with dental implants. An "Intervention" is the occurrence of fractures and the different characteristics of fractured implants. The "Comparison" corresponds to not fractured implants. Finally, the "Outcome" was the influence of the implant characteristics on fracture incidence.

Inclusion criteria were as follows: English, prospective, retrospective randomized controlled clinical trials; the study should report at least one case of implant fracture, even if their aim was not to evaluate the fracture incidence.

Exclusion criteria consisted of duplicated, animal, cadaver and in vitro studies, isolated case reports, reviews, and studies that reported no cases of implant fractures.

The following information was collected from selected articles: number, gender and age of patients evaluated; number, material, diameter, length and installation region of both installed and fractured implants, and the loading period until implant fracture.

## Results

**Figure 1:**
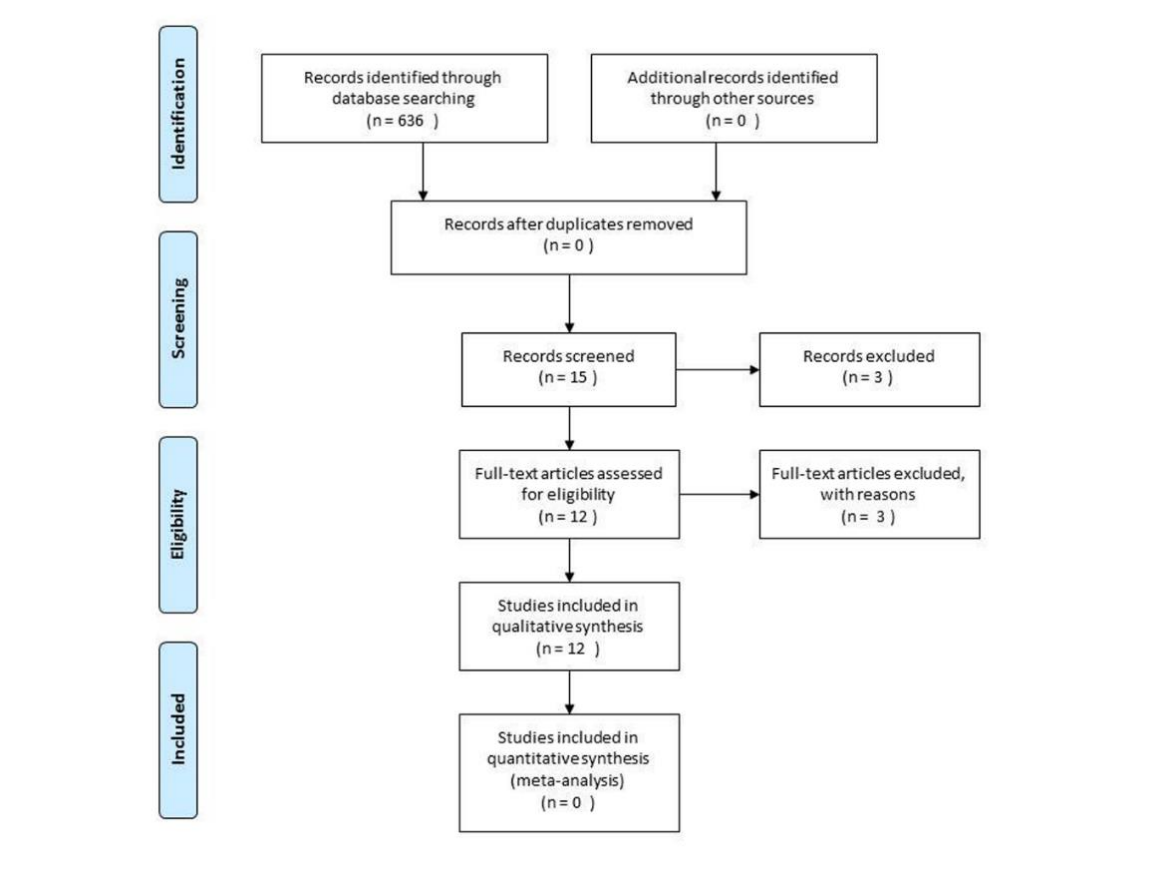
PRISMA flowchart.

A total of 632 studies were found with the search terms. By reviewing the titles and abstracts of each study, 15 were selected, of which one was excluded because it was not available for download at our institution and 2 were excluded after reading their full texts, for not fitting the inclusion criteria. Thus, 12 studies were selected to carry out this systematic review (Figure 1).

From the selected studies, 4 were prospective and 8 were retrospective. The number of patients evaluated was 594, with 354 women and 240 men, aged 17‒94 years, considering that one of the included studies did not provide such information.^14^ The follow-up of cases ranged from 1 to 20 years (Table 1).

**Table 1:** Demographic data of studies selected

**Study**	**Type of study**	**Follow-up**	**Number of patients evaluated**	**Female**	**Male**	**Age Range (Average Age)**
**Gargallo Albiol et al, 2008**	Retrospective	Minimum of 5 years	NR	NR	NR	NR
**Antoun et al, 2012**	Retrospective	17.6 months	44	32	12	51 a 94 - (70)
**Cha et al, 2013**	Retrospective	5 years	120	63	57	18.8 a 81.1 - (47)
**Eccellente et al, 2011**	Prospective	26.7 months	45	18	27	43 a 76 - (60)
**Eliasson et al, 2010**	Prospective	5 years	29	13	16	NR - (65)
**Gahlert et al, 2013**	Retrospective	Up to 3 years	79	43	36	19 a 85 - (52,2)
**Grant et al, 2009**	Retrospective	2 years and 1 month	124	89	35	18 a 80 - (56)
**Lekholm et al, 2006**	Retrospective	20 years	17	8	9	43 a 87 - (68)
**Misje et al, 2013**	Retrospective	12 to 15 years	18	11	7	17 a 41 - (NR)
**Šćepanović et al, 2012**	Prospective	1 year	30	16	14	45 a 63 - (NR)
**Wahlstrom et al, 2010**	Retrospective	Average of 5 years (40 to 84 months)	46	33	13	36 a 84 - (59)
**Preoteasa et al, 2014**	Prospective	3 years	23	13	10	52 a 76 – (62)
**Total**		1 to 20 years	594	354	240	17 a 94

NR: Not reported

Tables 2 and 3 show data regarding the installed implants and implants fractured for each study selected: number of implants, material, diameter, length, region (maxillary/mandibular) and loading time until fracture. It can be observed that not all studies provided full information, which was designated with "NR" (Not Reported).

**Table 2:** Number, material, diameter and length of implants installed and fractured

**Study**	**Implants installed**	**Implants fractured**	**Implant material**	**Diameter of implants installed (number of implants)**	**Diameter of implants fractured (number of implants)**	**Length of implants installed (number of implants)**	**Length of implants fractured (number of implants)**
**Gargallo Albiol et al, 2008**	1500	21	Titanium	NR	3.75 mm (20); 4 mm (1)	NR	10 to 15 mm
**Antoun et al, 2012(2)**	205	1	Titanium	3.35 mm (2) 3.75 mm (15) 4 mm (185) 5 mm (3)	NR	10 mm (3); 11.5 mm (13); 13 mm (86); 15 mm (103)	NR
**Cha et al, 2013**	136	11	Titanium	4 mm (136)	4 mm (11)	11/13 mm (124)	NR
**Eccellente et al, 2011**	180	1	Titanium	3.5 mm (153); 4.5 mm (27)	3.5 mm (1)	9.5 mm (3); 11 mm (18); 14 mm (92); 17 mm (1)	17 mm (1)
**Eliasson et al, 2010**	168	3	Titanium	3.75 mm (168)	3.75 mm (3)	10 mm (4); 13 mm (33); 16 mm (131)	NR
**Gahlert et al, 2013**	170	13	Zirconia	3.25 mm (59); 4 mm (82); 5 mm (29)	3.25 mm (12); 4 mm (1)	10 to 13 mm	NR
**Grant et al, 2009**	335	1	Titanium	3.5 mm (42); 4.3 mm (212); 5 mm (75); 6 mm (6)	NR	8 mm (335)	8 mm (1)
**Lekholm et al, 2006**	69	4	Titanium	4 mm (69)	4 mm (4)	7 mm (38%); 10 mm (33%); 13/15 mm (29%)	NR
**Misje et al, 2013**	22	1	Titanium	3.75 mm (21) 3.25 (1)	3.75 mm (1)	15 mm (13); 13 mm (8) 10 mm (1)	10 mm (1)
**Šćepanović et al, 2012**	123	3	Titanium	1.8 mm (123)	1.8 mm (3)	13 mm (123)	13 mm (3)
**Wahlstrom et al, 2010**	116	4	Titanium	NR	NR	NR	NR
**Preoteasa et al, 2014**	110	2	Titanium	1.8 mm (NR) 2.1 mm (NR) 2.4 mm (NR)	NR	10 mm (NR) 13 mm (NR) 15 mm (NR) 18 mm (NR)	NR
**Total**	3134	65					

NR: Not reported

**Table 3:** Region (maxillary/mandibular) of implants installed and fractured, and loading period until fracture occurred

**Study**	**Number of implants installed (Maxilla/Mandible)**	**Number of fractures per region (Maxilla/Mandible)**	**Loading period until fracture**
**Gargallo Albiol et al, 2008**	NR (Maxilla) NR (Mandible)	NR (Maxilla) NR (Mandible)	2 years (2) 3-4 years (16) 5-6 years (1) 9 years (2)
**Antoun et al, 2012(2)**	78 (Maxilla) 124 (Mandible)	0 (Maxilla) 1 (Mandible)	21 months
**Cha et al, 2013**	70 (Maxilla) 66 (Mandible)	NR (Maxilla) NR (Mandible)	6 months (1); 10 months (1); 1 year (2); 2 years (7)
**Eccellente et al, 2011**	180 (Maxilla) 0 (Mandible)	1 (Maxilla) 0 (Mandible)	6 months
**Eliasson et al, 2010**	0 (Maxilla) 168 (Mandible)	0 (Maxilla) 3 (Mandible)	3 years (1); 4 years (2)
**Gahlert et al, 2013**	90 (Maxilla) 80 (Mandible)	NR (Maxilla) NR (Mandible)	NR
**Grant et al, 2009**	0 (Maxilla) 335 (Mandible)	0 (Maxilla) 1 (Mandible)	10 months
**Lekholm et al, 2006**	NR (Maxilla) NR (Mandible)	2 (Maxilla) 2 (Mandible)	2 years (1); 7 years (1); 17 years (2)
**Misje et al, 2013**	22 (Maxilla) 0 (Mandible)	1 (Maxilla) 0 (Mandible)	10 years
**Šćepanović et al, 2012**	0 (Maxilla) 123 (Mandible)	0 (Maxilla) 3 (Mandible)	Not loaded.
**Wahlstrom et al, 2010**	116 (Maxilla) 0 (Mandible)	4 (Maxilla) 0 (Mandible)	6.5 years (3); 3 years (1)
**Preoteasa et al, 2014**	36 (Maxilla) 74 (Mandible)	NR (Maxilla) NR (Mandible)	1 to 2 years
**Total**	760 (Maxilla) 970 (Mandible)		

NR: Not reported

Twelve studies reported 3134 implants installed, of which 94.6% were titanium and 5.4% were zirconia implants. Of the implants placed, 65 were lost by fracture, representing an incidence of 2.0%. Among the 65 fractured implants, 20% were zirconia and 80% were titanium implants (Table 2).

Considering the studies that provided complete information about installation region, 760 implants were installed in the maxilla and 970 in the mandible. The implant loading period until fracture ranged from "not loaded" to 17 years (Table 3).

Data about fractured implants were also evaluated considering the studies that provided information about both installed and fractured implants for the different variables evaluated, allowing a proper comparison (Tables 4 to 6).

Diameter of Implants Installed vs. Diameter of Im-plants Fractured

Seven studies provided information about diameters of both the installed and fractured implants (Table 2). Thus, 868 implants were reported with seven different diameters. The most commonly used diameter was 4 mm ‒ 287 implants, of which 17 fractured, corresponding to a fracture incidence of 5.9% (Table 4).

The installation of implants with diameters of 4.5 and 5 mm was less frequent (27 and 29 implants, respectively), followed by implants 3.25 mm in diameter (60 implants). The first two diameters showed no fracture cases, while the 3.25-mm implants presented a higher incidence of fracture compared to the total implants of the same diameter and to fracture incidence in other diameters (Table 4).

**Table 4:** Fracture incidence for each diameter, according to the studies that provided information about installed and fractured diameters

**Diameters installed**	**Total number of implants**	**Implants fractured (%)**
1.8	123	3 (2.4)
3.25	60	12 (20)
3.5	153	1 (0.6)
3.75	189	24 (12.7)
4	287	17 (5.9)
4.5	27	0 (0)
5	29	0 (0)
Total	868	57

### Lengths of Implants Installed vs. Lengths of Implants Fractured

From the 12 selected studies, only 4 provided the lengths of both installed and fractured implants (Table 2). In these 4 studies, 594 implants presented 8 different lengths. The most common was the 8-mm-long implant (56,4%); however, only one 8-mm-long implant fractured, corresponding to 0.3% of fracture in relation to total implants of the same length (Table 5).

Regarding implants 10 and 17 mm in length, one implant was installed in each category and both fractured, corresponding to 100% fracture of implants with such lengths (Table 5).

**Table 5:** Fracture incidence for each length, according to the studies that provided information about installed and fractured lengths

**Lengths installed**	**Implants installed**	**Implants fractured**
8	335	1 (0.3%)
9.5	3	0
10	1	1 (100%)
11	18	0
13	131	3 (2.3%)
14	92	0
15	13	0
17	1	1 (100%)
Total	594	6

### Regions of Installed and Fractured Implants

Six studies allowed comparison of regions of installation and fracture (Table 3). Most implants were installed in the mandible (61.3%) (Table 6). However, the highest incidence of fractures occurred in implants installed in the maxilla (1.5%) (Table 6).

**Table 6:** Fracture incidence in maxilla and mandible, according to the studies that provided information about regions of installed and fractured implants

**Region (Maxilla/Mandible)**	**Installed**	**Fractured**
Maxilla	396 (38.7%)	6 (1.5%)
Mandible	627 (61.3%)	8 (0.8%)
Total	1023	14

The implant loading period until fracture was reported by 11 studies (Table 3). Of these, 50 fractures were observed, with 38.5% occurring between 3 to 4 years of implants in function (Table 7).

**Table 7:** Loading period until fracture and number of implants fractured in each period

**Loading period until fracture**	**Implants fractured**
**Before loading**	3
**6 months**	2
**10 months**	2
**1–2 years**	15
**3–4 years**	20
**5–6 years**	1
**6.5 years**	3
**7 years**	1
**9–10 years**	3
**17 years**	2
Total	52

## Discussion

Of all the mechanical complications, the implant fracture is considered the most frustrating and might occur after a certain period in function. Literature reports a large variation (0% to 3.45%) in the incidence of implant fractures,,^5,15^ although the studies that have reported 0% of fracture incidence exhibit relatively small sample sizes and short periods of follow-up.^16,17^ A 10-year follow-up study, with 1618 implants, reported an 0.8% fracture incidence, with only 13 implants fractured.^18^ In contrast, Adell et al^19^ evaluated 1997 Branemark implants and reported a relatively high fracture rate of 3.45% over a period of 15 years of follow-up. Balshi^9^ published data from 4045 implants and reported a 0.2% fracture rate over 5 years. This systematic review reported a fracture incidence of 2% in the studies selected, a value that fits the range demonstrated in the literature.

From the 65 fractured implants found in the studies selected for this review, 20% were zirconia (n=13) and 80% (n=52) were titanium implants. The reasons for titanium implants’ fracture are well described in the literature,^9,20-22^ with overload identified as the major cause,^5,23^ being first attributed to a progressive fatigue until implant loses the appropriate strength to maintain its integrity, culminating in a catastrophic failure.^3^ Factors such as implant design, manufacturing defects and lack of a passive fit of prosthesis can also be associated with fractures.^20^ The absence of periodontal ligament which is present in natural teeth and direct bone apposition to the implant do not allow its movement when subjected to occlusal loads and may result in excessive stress, leading to different mechanical failures, among them, the implant fracture.^24^

Regarding zirconia implants, Gahlert et al^20^ assessed 119 implants and reported 13 lost by fracture. Macroscopic and microscopic analyses of these fractures have shown that overload, implant design, and surface microcracks due to manufacturing process were also the main reasons for zirconia implants’ fracture.^25^ Zirconia milling process might result in imperfections and microcracks^26,27^ that can influence the fracture resistance and material reliability.^20,26-28^

Regarding zirconia implants, Gahlert et al^20^ assessed 119 implants and reported 13 lost by fracture. Macroscopic and microscopic analyses of these fractures have shown that overload, implant design, and surface microcracks due to manufacturing process were also the main reasons for zirconia implants’ fracture.^25^ Zirconia milling process might result in imperfections and microcracks^26,27^ that can influence the fracture resistance and material reliability.^20,26-28^

### Diameter

In this systematic review, the highest incidence of fractures in terms of diameter occurred in implants 3.25 mm in diameter (20%) (Table 4), which are considered small-diameter implants. In a recent systematic review, Klein et al^29^ assessed the success of narrow-diameter implants. The authors showed survival rates between 90.9% and 100% for implants <3 mm in diameter, while for diameters between 3.0 and 3.25 mm, survival rates ranged from 93.8% to 100%.^30,31^

It has been shown that a reduced implant diameter might reduce the osseointegration surface and compromise the mechanical conditions in the implant body, abutment and screw components.^32,33^ Moreover, narrow-diameter implant assemblies are more prone to mechanical failure because of their compromised fatigue, with the effects of magnitude of force and angulation being of greater clinical significance when narrow-diameter implants are used.^5,33^

Although narrow-diameter implants show greater propensity to failures, a study by Šćepanović et al^34^ showed that 1.8-mm-diameter implants exhibited a low fracture incidence (2.4%) (Tables 2 and 4), which could be justified by the fact that most of the implants evaluated were splinted to receive the prosthesis. On the other hand, it is observed that in this review, 12 fractured implants with 3.25 mm belong to the study of Gahlert et al,^20^ (Table 2), which, in addition to being zirconia implants, were installed to support one-unit prostheses. A study showed better sharing of occlusal loads and distribution of stress with splinted versus individually restored implant designs.^35^

Literature describes that narrow-diameter implants should be used with specific indications.^30^ These implants are offered by almost all implant manufacturers and are designed specifically for restricted interdental spaces, mandibular incisors and maxillary lateral teeth.^32,36^ In contrast to what is described, a long survival period might be expected for reduced diameter implants, as long as the number of implants used is sufficient to support well planned prostheses.

### Length

The literature is heterogeneous about the survival rates regarding implant length, as well as the definition of short implants, which varies from 4 to 11 mm, with some studies considering standard-length implants as implants with lengths of ≥10 mm.^37^ Winkler et al^38^ demonstrated a survival rate of 66.7% for short implants (7 mm) and 96.4% for long implants (16 mm) after 6 months in situ.^1^ Another recent study detected similar survival rates of short and long implants.^39^ Annibali et al^40^ published a systematic review including clinical studies of short implants (<10 mm) placed in the maxilla and mandible (6193 implants in 3848 participants) and reported an overall cumulative survival rate of 99.1%, while Srinivasan et al^37^ reported survival rates ranging from 92.2% to 100% for implants <8 mm in length.

In this review, short implants (8 mm) showed only 0.3% of fractures compared to the total number of implants (Table 5), while longer implants (10 and 17 mm) presented 100% of fracture. Although many studies have demonstrated higher failure rates for short implants, recent reports show survival rates of these implants similar to longer implants.^41^ In 2006, Misch et al^41^ published a literature review of failure rates associated with dental implants <10 mm in length in the posterior regions of partially edentulous patients undergoing placement from 1991 to 2003. They reported that among 2837 short implants, survival rate was 85.3%. Furthermore, they and other authors have shown that failures are independent of implant length, with no clear linear relationship between the length and implant survival.^40,42^

It should be noted that data extracted from studies on this topic are limited and inconclusive. Since, in this systematic review, only one implant in both the 10- and 17-mm lengths was found, this is not sufficient to draw conclusions regarding the success/failure of long implants. Still, few studies provided data about the length of implants installed and fractured. Only 4 of the selected studies provided this information, where from 594 implants placed, we have data from only 6 of the fractured implants (Table 5).

### Installation Region (Maxilla/Mandible)

In this systematic review, failures were interestingly observed more frequently in the maxilla than in the mandible (1.5% to 0.8%, respectively) (Table 6), similar to a study by Srinivasan.^38^ In a long-term multicenter study, Adell et al^19^ reported a cumulative implant fracture rate of less than 5% in both the maxilla and mandible in a period of 10 to 15 days, except for one study group where fracture rate reached 16% in the maxilla. It can be explained by the fact that shape and bone density are important factors for implants survival.^33^ The mandible is a cortical bone, while the maxillary bone is trabecular and less mineralized, which might compromise primary implant stability and lead to future failures.^32^

A systematic review reported an implant fracture rate of 0.5% after 5 years (Jung et al, 2008).^15^ In the study of Adell et al,^19^ an incidence rate of 1.0‒3.5% for implant fracture was observed, with most fractures also occurring after 5 years of clinical function. However, in this systematic review, the largest number of fractures was reported in a range of 3‒4 years (20 fractures, Table 7), which can be attributed to the fact that from the 12 selected studies, 6 underwent follow-ups shorter than 5 years (Table 1).

The studies selected exhibited a broad diversity in terms of implant length and diameter, location of installation, study design and observation period. Furthermore, the studies showed variations related to unspecified dropouts, specific time of fracture and method of statistical analysis. These factors deemed it impossible to systematically compare the reviewed publications with one another; which was a similar finding in an earlier published review. Hence, a descriptive, but nevertheless structured and methodologically solid analysis was performed in this review.^37^

As could be observed, some studies in the literature do not provide necessary information to establish a relationship between the different parameters of implants and the incidence of fractures. However, from the above, we consider that although the incidence of implant fractures is relatively low, this failure can be avoided taking into consideration the different implant characteristics. The indication of type, diameter and length of an implant and bone quality in the region that will receive it should be studied and evaluated precisely for each specific case.

## Authors’ Contributions

AMA and ASN were responsible for data collection and interpretation, preparation of the English manuscript and data interpretation. DMS and MCG were responsible for the project and interpretation and provided advice and revised important intellectual content. FPC was responsible for data interpretation, revision of important intellectual contente and final review.

## Funding

This study had no funding source.

## Conflict of Interests

The authors declare no conflict(s) of interest related to the publication of this work.
